# Comparison of the Therapeutic Effect of the Mini-Open Incision and Conventional Open Neurolysis of the Median Nerve for Carpal Tunnel Syndrome

**DOI:** 10.1155/2022/4082618

**Published:** 2022-10-21

**Authors:** Feng Hu, Liang Lu, Jianxue Zeng, Duoyu Li, Bin Liu

**Affiliations:** Department of Orthopedics, The First Affiliated Hospital of USTC Anhui Provincial Hospital, No. 17 of Lujiang Road, Hefei, China

## Abstract

**Objective:**

The aim of this study was to compare the therapeutic effects of the mini-open incision and conventional open surgery for carpal tunnel syndrome (CTS).

**Methods:**

The clinical data of 52 patients with CTS treated at the First Affiliated Hospital of the University of Science and Technology of China from October 2020 to February 2022 were retrospectively analyzed. The patients were divided into the conventional open surgery group (28 cases) and the mini-open incision group (24 cases) according to different surgical incisions applied. The incision length, operation time, time until postoperative return to work, and complications were observed in the two groups. The Visual Analog Scale (VAS) for pain at one day, one month, and three months after surgery and the Boston Carpal Tunnel Questionnaire scores before, at one month, and at three months after surgery were compared between the two groups.

**Results:**

The incision length, operation time, and time until return to work in the mini-open incision group were all shorter than those in the conventional open surgery group (2.58 ± 0.35 vs. 7.32 ± 0.61 cm, 18.67 ± 2.62 vs. 29.46 ± 3.42 min, and 5.33 ± 1.40 vs. 13.86 ± 2.70 d, respectively), and differences were statistically significant (*P* < 0.05 in all). The VAS scores in the mini-open incision group were lower than those in the conventional open surgery group at one day and one month after surgery, while the difference in the VAS scores at three months after surgery was not statistically significant between the two groups. There was no statistically significant difference in neurological recovery between the two groups at postoperative follow-ups (*P* > 0.05). The incidences of postoperative scar hyperplasia and scar pain were higher in the conventional open surgery group than those in the mini-open incision group, and differences were statistically significant (*P* < 0.05 in both).

**Conclusion:**

Mini-open incision surgery for CTS was a safe and reliable procedure with a precise therapeutic effect, minimal surgical trauma, and high postoperative comfort for patients and could achieve enhanced recovery. *Trial Registration*. This trial is registered with ChiCTR2200064631.

## 1. Introduction

Carpal tunnel syndrome (CTS) is the most common clinical peripheral nerve entrapment syndrome and is a solitary neurological disorder that occurs when the median nerve is compressed as it passes through the inelastic carpal tunnel. Repeated flexion and extension of the wrist joint during daily life and work may lead to thickening of the transverse carpal ligament and consequent compression of the median nerve [1]. Increased content in the carpal tunnel, such as synovial hyperplasia, ganglion cysts, or schwannoma, may also lead to median nerve compression. The early symptoms of CTS are abnormal sensation and/or numbness in the median innervation area (thumb, index finger, middle finger, and the radial half of the ring finger), with nocturnal finger numbness being the first symptom in most patients. As the disease worsens, patients may develop atrophy of the thenar muscle and limited opposition of the thumb function. Sleep quality, the ability to perform daily activities, and mental health are seriously affected in patients with CTS [2].

In the early stages of CTS, conservative treatment may be considered, including relief of inflammation by physical therapy and using hand splint to limit the activity of the wrist [3]. Paraffin treatment is also widely used as a physical therapy method for the treatment of CTS. According to the study performed by Ordahan and Karahan, paraffin treatment increases the recovery in functional and electrophysiological parameters [4]. If strictly conservative treatment is ineffective, it may lead to irreversible damage due to compression degeneration of the median nerve, and surgery is recommended for these patients. Although the conventional neurolysis of the median nerve gives rise to complete decompression, surgical incision is long, and postoperative recovery is protracted. Some patients have limited wrist movement due to scar hyperplasia and pain after surgery, and serious cases require secondary surgery [5].

In recent years, the neurolysis of the median nerve under wrist arthroscopy or endoscopy has become a popular topic. However, this technique has a long learning curve, and it is easy to injure the median nerve and its branches during surgery, which is an undesirable outcome [6, 7]. The effect of cutting the transverse carpal ligament through a mini-open incision for the neurolysis of the median nerve is not only definite but also causes less trauma and incision scarring and leads to rapid recovery after surgical treatment [8].

To further investigate the therapeutic effects, advantages, and disadvantages of the mini-open incision surgery and conventional open surgery, the efficacy of the two surgical approaches were compared in the present study by analyzing the clinical data of 52 patients with CTS who underwent surgical treatment in the subject hospital.

## 2. Material and Methods

### 2.1. Inclusion and Exclusion Criteria

The inclusion criteria were as follows: (1) patients who met the diagnostic criteria for CTS; (2) patients whose symptoms were not relieved, recurrent, or even aggravated after more than 3 months of strict conservative therapy; and (3) patients with slowed median nerve conduction confirmed by the electrodiagnostic test, thickening of the transverse carpal ligament revealed by wrist color Doppler ultrasonography, and median nerve compression.

The exclusion criteria were as follows: (1) patients with CTS combined with peripheral neuropathy, such as diabetes mellitus, cervical spondylosis, or hypothyroidism; (2) patients with pathological changes, such as intracarpal tunnel masses, gout stones, or rheumatoid synovitis; and (3) patients with previous brachial plexus injury, wrist trauma, wrist deformity, or steroid injection in the wrist.

### 2.2. General Characteristics

The present study was a retrospective one. Fifty-two patients with CTS admitted to the First Affiliated Hospital of the University of Science and Technology of China between October 2020 and February 2022 were enrolled. They were divided into the conventional open surgery group (the open carpal tunnel release (OCTR)) and the mini-open incision group (the modified mini-open incision carpal tunnel release (MICTR)) according to the shape of the surgical incision. There were 28 cases in the conventional open surgery group, with 5 males and 23 females aged 44–74 years, with an average age of 55.2 years. There were 24 cases in the mini-open incision group, with 5 males and 19 females, aged 30–69 years, with an average age of 53.8 years. The present study complied with the Declaration of Helsinki, and all patients signed the informed consent form for the procedure.

### 2.3. Surgical Methods

#### 2.3.1. Conventional “S”-Shaped Open Carpal Tunnel Release on the Wrist

**Figure 1:**
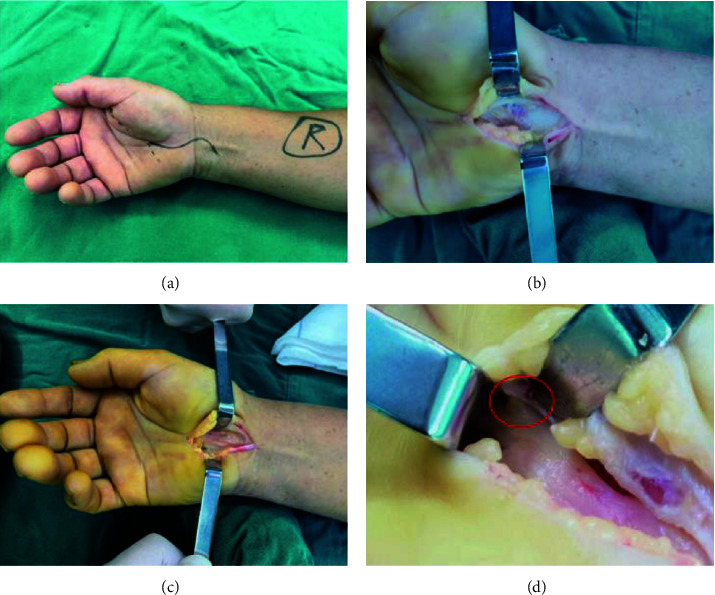
An “S”-shaped incision was made across the wrist of a length of approximately 6–8 cm (the dotted line is a Kaplan cardinal line and the solid line is an incisional site). (a) The incised skin was retracted, subcutaneous tissue and palmar aponeurosis were bluntly dissected using vascular forceps, and the transverse carpal ligament was exposed. (b) The ligament was cut with a surgical blade. (c) Exploration of the recurrent branch of the median nerve (d).

Before surgery, the affected hand, wrist, and forearm were cleaned with povidone-iodine solution. A pneumatic tourniquet was used. Under nerve block anesthesia, the skin was incised, and subcutaneous tissue and palmar aponeurosis were bluntly dissected. Under direct vision, the transverse carpal ligament was incised, and the median nerve was released Figure 1. If severe epineurial fibrosis was found during the operation, we would perform external epineurotomy. The tourniquet was loosened, and the skin was sutured layer by layer after complete hemostasis.

#### 2.3.2. Transwrist-Modified Mini-Open Incision Carpal Tunnel Release

**Figure 2:**
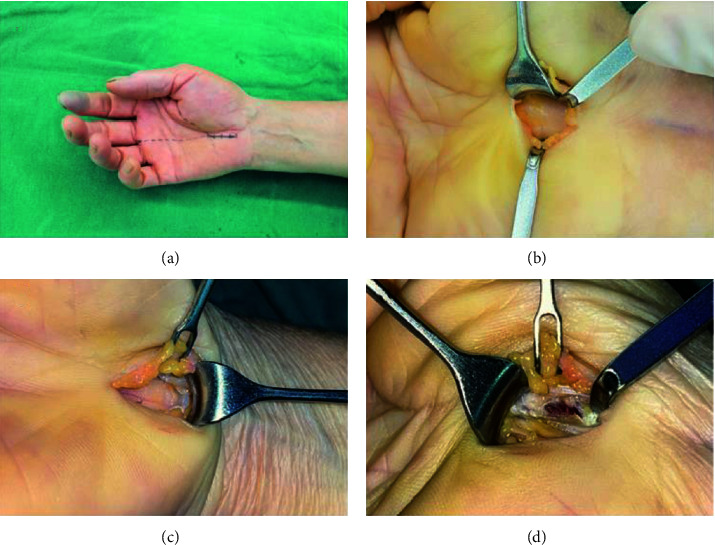
A longitudinal incision placed in the radial border of the ring finger line, which begins about 1 cm to the distal flexor wrist crease (dotted lines are the Kaplan cardinal line and radial side ring finger and the solid line is an incisional site). (a) The incised skin was retracted with the help of a miniretractor, subcutaneous fat tissue was dissected, and the ligament was cut with a surgical blade. (b) The wrist joint was flexed palm forward, and the ligament was released to the level of the distal flexor wrist crease. (c) The wrist joint was extended dorsally, and the branch of the median nerve could be released under direct vision (d).

Under nerve block anesthesia, the surgical incision that varies from 2 cm to 2.5 cm was placed in the radial border of the ring finger line which begins about 1 cm to the distal flexor wrist crease (Figure 2). The skin was incised, and subcutaneous adipose tissue and palmar aponeurosis were bluntly dissected using vascular forceps until the transverse carpal ligament was exposed. The transverse carpal ligament was cut with a surgical blade, and its position at the median nerve was carefully identified to avoid accidental injury due to positional variation. A nerve stripper was inserted under the transverse carpal ligament to protect the median nerve, and the transverse carpal ligament was incised along the ulnar side of the median nerve under direct vision. The operation should be as close to the ulnar side as possible to avoid injury to the superficial palmar arch, palmar cutaneous branch, and recurrent branch of the median nerve. The wrist joint was flexed palm forward, the skin at the proximal end of the incision was pulled up with a retractor, and the wrist ligament was released to the level of the distal flexor wrist crease. Then, the wrist joint was extended dorsally, the skin at the distal end of the incision was pulled up with a retractor, and the transverse carpal ligament was released to the level of the branch of the median nerve, and the epineurium was released if necessary. The tourniquet was loosened with complete hemostasis. The skin was sutured intermittently after flushing.

### 2.4. Observation and Evaluation Indicators

The incision length and operation time were recorded in the two groups, and the time of postoperative return to work and complications (including median nerve branch injury, superficial palmar arch and adjacent tendon injury, surgical site infection, hematoma, scar hyperplasia, scar pain, and column pain) were statistically analyzed in the two groups. The Visual Analogue Scale (VAS) was adopted for pain scoring at one day, one month, and three months after surgery, and the Boston Carpal Tunnel Questionnaire score was used to evaluate neurological recovery before, at one month, and three months after surgery. At the last follow-up, the Kelly grading scale was used to evaluate the surgical efficacy. The grading scale rated surgical outcomes excellent (with complete relief of symptoms), good (with occasional and persisted mild symptoms), fair (with some persistent or distressing symptoms), and poor (with unchanged or worsened symptoms).

#### 2.4.1. Evaluation Indicators

 
*(1) VAS*. The Visual Analogue Scale was used as a pain assessment parameter. “0” indicated no pain/no numbness, whereas “10” indicated that there is enough pain/numbness to tolerate.
 
*(2) Boston CTS Questionnaire*. The Boston CTS Questionnaire was used to assess disease severity and functional status. It included 19 questions, in which 11 questions aimed to assess symptoms and 8 questions examined functional capacity. The mildest symptoms or best functional capacity were rated as 1 point, whereas the heaviest symptoms or worst functional condition were given 5 points in the responses. Average scores were obtained by dividing the total score obtained by the number of questions. Average scores were used in this study.
 
*(3) Kelly grading scale*. The Kelly grading scale was used to evaluate the outcome of surgery according to relief of symptoms. Excellent indicated complete relief of symptoms, good indicated persistence of occasional minor symptoms, fair indicated some constant or annoying symptoms, and poor indicated symptoms unchanged or worse.

### 2.5. Statistical Methods

The SPSS™ Statistics v20.0 software package was adopted for data processing. The measurement data were expressed as a mean ± standard deviation ($\bar{x}$ ± *s*), and the independent samples *t*-test was used for comparison between groups. The *χ*^2^ test was adopted for the countable data. A value of *P* < 0.05 was considered statistically significant.

## 3. Results

The differences in age and gender distribution were not statistically significant between the two groups (*P* > 0.05 in both) (Table 1).

The incision length, operation time, and time of return to work in the mini-open incision group were all shorter than those in the conventional open surgery group, and differences were statistically significant (*P* < 0.05 in all). The VAS scores in the mini-open incision group were lower than those in the conventional open surgery group at one day and one month after surgery, and differences were statistically significant (*P* < 0.05 in both). However, the difference in the VAS scores three months after surgery was not statistically significant between the two groups (*P* > 0.05) (Table 2).

The Symptom Severity Scale and Functional Status Scale scores of the two groups of patients at the 1-month and 3-month follow-up after the operation were lower than those before surgery, and differences were statistically significant (*P* < 0.05). However, there was no significant difference between the two groups at the same follow-up time point (Table 3).

At the last follow-up, the excellent and good rate by the Kelly grading scale in the mini-open incision group was 95.8%, while that in the open surgery group was 85.7%, and the difference was not statistically significant. In the open surgery group, five patients developed obvious scar hyperplasia after surgery, and two of them were accompanied by scar pain. In the mini-open incision group, one patient had mild scar hyperplasia without discomfort. No median nerve branch injury occurred in either group of patients (Table 4).

## 4. Discussion

According to the results of this study, although both groups achieved good results at 3 months after the operation, a significant reduction in hand pain, shorter duration of the surgical procedure, shorter period of time to return to work, and lower probability of scar hyperplasia were observed after mini-open incision carpal tunnel release, which is really similar to the results of the literature reviews that used mini-open incision approaches [9–11]. On the other hand, we did not experience any artery or nerve injury while using a mini-open incision approach, which was reported in several previous trials using a mini-incision technique [12].

CTS is one of the most common compressive neuropathies in clinical practice. In previous studies, it affected mainly the middle-aged population and mostly females [12–15]. In our study, CTS was more frequent in women (female/male: 4.2) and with a mean age of 54.6 years. For moderate to severe CTS, a complete cure mainly depends on cutting the transverse carpal ligament and releasing the median nerve. Surgical treatment of CTS aims to increase the space in the carpal tunnel by cutting off the transverse carpal ligament and then reduce the pressure on the median nerve [10]. There are several modalities of surgical treatment for CTS, and the most common approaches are open carpal tunnel release through a standard or limited incision. In recent years, the application of the endoscopic carpal tunnel release (ECTR)methods has been reported. Although some trials have reported a sooner recovery and back to work by ECTR, other studies have not shown significant differences between ECTR and open carpal tunnel release [14, 16, 17]. Furthermore, although ECTR may lead to less postoperative pain and local tenderness, the degree of this advantage seems to be modest [16]. The conventional OCTR method is the classic operation for the treatment of CTS with sufficient intraoperative exposure and complete decompression. However, it is also accompanied by certain complications, such as scar hyperplasia, scar pain, and neurovascular injury [18]. The present study found that compared with conventional open surgery, mini-open incision surgery could achieve the same nerve decompression effect and effectively relieve postoperative scar hyperplasia and symptoms of scar pain with a shorter operation time and sooner recovery. This is conducive to patients' enhanced recovery, which would enable them to return to society and work earlier.

In a previous study, it reported that a longitudinal incision is more effective in relieving symptoms and better functional outcomes than a transverse incision. [19] So the design of the incision is a key element affecting the effect of mini-open incision surgery. In our study, the incision was placed in the radial border of the ring finger line, which begins about 1 cm from the distal flexor wrist crease and is distal to the Kaplan baseline [19]. For patients who were obese, the incision would be appropriately extended to facilitate the exposure of the surgical field. However, it was recommended that the proximal end should not exceed the distal wrist crease to prevent postoperative scar hyperplasia from affecting the flexion and extension of the wrist joint in patients with scar constitution.

The recurrent branch of the median nerve, also known as the thenar branch, originates from the common first digital nerve and the trunk of the median nerve. Most of them arise from the distal end of the transverse carpal ligament and outside the carpal tunnel, and some run along the carpal tunnel or pass through the transverse ligament. Regardless of the relationship between the recurrent branch and the transverse carpal ligament, most of its origins and courses are on the radial side of the median nerve [20]. Therefore, when cutting the transverse carpal ligament, it should be conducted on the ulnar side of the median nerve. The recurrent branch of the median nerve should be explored in this incision but not as a routine exploration. The appropriate release might be given for patients with thenar atrophy or hypoesthesia in the thenar region. During the operation, the wrist joint was extended dorsally at 45°, and subcutaneous tissue was retracted with a miniature deep skin retractor, so the recurrent branch of the median nerve could be more clearly exposed. If the patient has pain in the thenar area and thenar muscle atrophy before surgery, the recurrent branch of the median nerve should be explored during the operation.

It was suggested that post-CTS column pain is associated with injury to the palmar cutaneous branch of the median nerve [21], which originates at the intersection of the distal wrist crease and the extension line of the middle axis of the middle finger and runs on the radial side of the median nerve with many distributions in the subcutaneous tissue of the midpalmar and thenar regions [22]. Therefore, after incision of the skin, blunt dissection of the subcutaneous tissue and palmar fascia with the adoption of vascular forceps was recommended until the transverse carpal ligament was exposed. Sharp incisions in subcutaneous tissue with a razor blade should be avoided to prevent injury to the palmar cutaneous branch of the median nerve and its branches.

## 5. Conclusion

In summary, the mini-open incision surgery in the treatment of CTS had the advantages of a small incision, quick recovery, mild postoperative pain, and good scar appearance. It is a safe and effective surgical approach, and with mastery of the local anatomy and surgical details, it could effectively reduce the incidence of nerve injury.

## Figures and Tables

**Table 1:** Comparison of the general characteristics between the two groups of patients.

Groups	Number of cases	Age (year)	Gender (number of cases)		BMI
			Male	Female	
Small incision group	24	53.83 ± 9.32	5	19	23.58 ± 3.26
Open surgery group	28	55.21 ± 6.32	5	23	23.32 ± 3.12
Statistics		*t* = −0.633	*χ* ^2^ = 0.074		*t* = 0.296
*P*		>0.05	>0.05		>0.05

**Table 2:** Comparison of the intraoperative and postoperative conditions between the two groups of patients.

Groups	Incision length	Operation time	Time of postoperative return to work	VAS		
				One day after surgery	One month after surgery	Three months after surgery
Small incision group	2.58 ± 0.35	18.67 ± 2.62	5.33 ± 1.40	1.67 ± 0.81	0.71 ± 0.62	0.25 ± 0.44
Open surgery group	7.32 ± 0.61	29.46 ± 3.42	13.86 ± 2.70	3.64 ± 0.78	1.82 ± 0.81	0.32 ± 0.48
Statistics						
*P*	<0.05	<0.05	<0.05	<0.05	<0.05	>0.05

**Table 3:** Comparison of the preoperative and postoperative BCTQ scores between the two groups of patients.

Groups	Before operation	One month after surgery	Three months after surgery
SSS score			
Small incision group	3.33 ± 0.76	2.16 ± 0.70	1.54 ± 0.51
Open surgery group	3.46 ± 0.88	2.00 ± 0.72	1.46 ± 0.50
*T*	0.57	0.84	0.55
*P*	>0.05	>0.05	>0.05

FSS score			
Small incision group	2.96 ± 0.85	1.42 ± 0.50	1.29 ± 0.69
Open surgery group	3.07 ± 0.81	1.53 ± 0.57	1.17 ± 0.39
*T*	0.49	0.78	0.74
*P*	>0.05	>0.05	>0.05

**Table 4:** Comparison of the Kelly grades and incidences of complications after the operation between the two groups of patients.

Groups	Kelly grade				Rate of excellentand good (%)	Scar hyperplasia		Scar pain	
	Excellent	Good	Fair	Poor		With	Without		
Small incision group	21	2	1	0	95.8	1	23	0	24
Open surgery group	20	4	3	1	85.7	7	21	5	23
Statistics					*χ* ^2^ = 1.523	*χ* ^2^ = 4.309		*χ* ^2^ = 4.742	
*P*					>0.05	<0.05		<0.05	

## Data Availability

The datasets used and/or analyzed during the current study are available from the corresponding author on reasonable request.
